# An exploration of knowledge, attitudes and advice given by health professionals to parents in Ireland about the introduction of solid foods. A pilot study

**DOI:** 10.1186/1471-2458-10-201

**Published:** 2010-04-21

**Authors:** Claire Allcutt, Mary-Rose Sweeney

**Affiliations:** 1 UCD School of Public Health and Population Science, University College Dublin, Dublin, Republic of Ireland; 2 School of Nursing, Dublin City University, Dublin, Republic of Ireland

## Abstract

**Background:**

For the purposes of this paper "weaning is defined as the introduction of the first solid foods to infants". Global recommendations by the World Health Organisation (WHO) recommend that all infants be exclusively breast-fed for the first six months of life. No global recommendations have been made for formula fed infants. In Europe it is recommended that weaning foods should be introduced between 18 weeks and 26 weeks regardless of whether infants are breast or formula fed. In the United Kingdom it is recommended that solids be introduced at around six-months for both breast and formula fed infants. In Ireland official guidelines recommend that breast fed infants should be introduced solids at 6 months of age while for formula fed infants the recommendation is for 4 months. The disparity between these global, European, UK and local recommendations may be a source of confusion for parents and health care professional based in Ireland. Emerging evidence suggests that babies in Ireland are given solid foods before the recommended age but there has been little investigation of the weaning advice provided by health professionals. Since community health professionals have routine parent interactions in the pre-weaning and early-weaning period and hence are in a unique position to positively influence parental weaning decisions, this study aimed to explore their knowledge, attitudes and advice practices about weaning.

**Methods:**

A mixed-methods approach was used for the research, commencing with a multi-disciplinary focus group to guide and develop a questionnaire. Questionnaires were then distributed in a postal survey to General Practitioners (GPs) (*n *179), Practice Nurses (PNs) (*n *121), Public Health Nurses (PHNs) (*n *107) and Community Dieticians (CDs) (*n *8).

**Results:**

The results indicate varying levels of knowledge of official weaning recommendations and a variety of advice practices. CDs and PHNs acknowledged a clear role in providing weaning advice while demonstrating high confidence levels in providing this advice. However, 19% of PNs and 7% of GP respondents did not acknowledge that they have a role in providing weaning advice to parents; even though Health Service Executive (HSE) written literature given to parents states that they should seek information from PNs and GPs.

**Conclusion:**

Small pockets of misinformation about the introduction of solid foods persist amongst health professionals which may lead to inconsistent advice for parents. Further research is needed.

## Background

The first months of an infant's life are characterised by rapid growth and development, with a corresponding period of parental learning and upheaval. Much early parental anxiety and uncertainty concerns infant feeding, with new parents seeking advice from family and friends, from books and the media, as well as from the health professionals who have responsibility to provide evidence-based, best practice advice. There is much concern, confusion and controversy around the introduction of solid foods due to changing guidelines and evidence about the health implications of the type and timing of solid foods.

Weaning is defined as the introduction of the first solid foods to infants. It is widely believed that there are significant health implications from the introduction of inappropriate solid foods to infants during weaning, including the risk of eczema, asthma, allergy and obesity. Prior to 2001 the World Health Organisation (WHO) recommended that infants be exclusively breast fed for 4-6 months [1]. However after a systematic review published in 2002 [2] they amended this with the global recommendation that solid foods should not be introduced before 6 months of age for breast-fed infants, however they did not specify any age for formula-fed infants. This systematic review was recently updated [3] with the group endorsing those previous recommendations of exclusive breastfeeding for the first six months of life in developing and developed countries with the caveat that individual infants should still be managed individually to prevent any adverse outcomes.

In Europe the European Society for Paediatric Gastroenterology, Hepetology, and Nutrition (ESPGHAN) committee position paper's review of practices in Europe in 2008 [4] concluded that complementary feeding should not be introduced before 17 weeks or after 26 weeks and further went on to make recommendations about food groups and when they should be introduced. However while acknowledging that theoretical benefits might accrue from separate recommendation for breast and formula fed infants they concluded that this would present considerable practical difficulties and therefore is undesirable.

In the UK, the six month recommendation of the WHO was endorsed by the Scientific Advisory Committee on Nutrition (2003) [5] stating that there should be flexibility in the advice given. After a period of heated discussion, in 2004 the United Kingdom (UK) Department of Health [6] decided on a recommendation that solids should be introduced at around six-months for both breast fed and bottle-fed infants.

In Ireland in 2003 the Department of Health and Children (DoHC) [7] endorsed the WHO guidelines for exclusive breastfeeding until 6 months, and changes were made to parental infant feeding literature produced by the newly formed Health Service Executive (HSE). However they did not make changes to the recommendations for formula fed infants. The current weaning recommendations in Ireland are that solid foods, except in special circumstances, should be introduced into an infant's diet at around 26 weeks of age for a breast-fed infant and not before 18 weeks for a formula-fed infant. Disparity between these global, European and local recommendations in Ireland may be the cause of some confusion for both parents and health service providers based there.

Contemporary research evidence suggests that babies in Ireland are given solid foods before the recommended age [8] and a number of studies have shown a link between the early introduction of solids and health risks, such as food intolerance, excess weight gain and Diabetes. Wilson *et al*, [9] reported that introducing solids before 15 weeks is associated with an increased likelihood of respiratory illness, particularly wheeze and persistent cough. Foote and Marriott [10] expressed concern that early solids might cause immune sensitisation and potential stress on the kidneys due to a high solute load from some weaning foods and Morgan *et al *[11] suggest that giving solids to pre-term infants before 17 weeks increases risk of eczema. More recently in a birth cohort study, on the timing of solid food introduction and its relationship to eczema, asthma, allergic rhinitis and food and inhalant sensitization at the age of six years [12] the authors found no evidence supporting a delayed introduction of solids beyond 4-6 months for the prevention of asthma, allergic rhinitis and food and inhalant sensitization at the age of six years. For eczema the results were conflicting however.

In a subsequent paper, late introduction of solid foods was in fact associated with increased risk of allergic sensitization of food (specifically oats and eggs) and inhaled allergens (specifically potatoes and fish) [13], however the authors stated a limitation of the study being that subjects were selected on the basis of HLA-conferred susceptibility to Type 1 Diabetes which may impact on the generalisability of the findings. Other papers have focused on the potential for nutrient deficiencies from delayed onset of weaning [14,15].

The Food and Nutrition guidelines for pre-school services 2004 [16] makes recommendations for the order and timing of first foods inclusive of stage 1 (introduction of complementary foods), stage 2 (over a 6 month period) and stage 3 (over a 9-12 month period). The general principles of this include commencing at stage one with gluten free cereals, pureed fruits and vegetables and iron rich foods such as pureed meats, progressing to stage 2 with a wider range including eggs, gluten containing cereals, cheese, and small amounts of cows milk and then stage 3 where most family foods are considered safe with the exception of high salt, sugar and nuts (until age 3).

While much research has focused on the actual recommendations and the issue of late/versus early introduction of solids and the impact on health there has been little investigation of the weaning advice provided by health professionals routinely caring for infants during this important period in their development, even though a recent paper suggests that the influence of health professionals on infant weaning practices has the potential to be as great as cultural values or material resources [17]. This is echoed in the findings of Ewing and Green [18] who demonstrate the significance of the health visitor in the UK in giving weaning advice. A review of the published literature sourced a number of studies in this area in the UK [19,20,11]. The findings of these studies were consistent in that they found that recommendations were varied and knowledge inconsistent. Our search did not source any studies conducted in the Republic of Ireland. The aim of this pilot study then was to explore knowledge and advice practices of health professionals working in Ireland with responsibility for advising parents on infant weaning.

## Methods

This exploratory study employed both qualitative and quantitative methods. The qualitative component was a multidisciplinary focus group, which preceded and informed the quantitative component, a postal questionnaire. The focus group was comprised of eight participants, two of each from the representative professions. The purpose of the focus group was to develop and guide the postal questionnaire.

The questionnaire design evolved from the literature, expert opinion and the focus group. The questionnaire was modified following a small pilot study, leading to changes in the format of the questionnaire and minor alterations in the wording of questions, before being distributed by post to the research population.

The research population comprised Health Professionals who regularly engage with parents during the pre weaning and weaning period, namely General Practitioners (GPs) (n = 179), Practice Nurses (PNs) (n = 121), Public Health Nurses (PHNs) (n = 107) and Community Dieticians (CDs) (n = 8). (There are only 8 community based dietician working in the geographical area under study, hence the relatively smaller sample).

Purposive sampling was used for the focus group and the sample for the postal survey which included health professionals who work with parents in a geographical area serving a patient population of around eight thousand infants each year. 415 questionnaires were sent with a single exclusion criterion that professionals who do not routinely work with young families should identify themselves and were subsequently excluded from the study.

### Data collection instrument and analysis

The focus group was audio-taped and the recording was transcribed immediately following the focus group. The transcription was analysed by identifying themes that had arisen during the discussions.

For the quantitative study we did not source any previously-validated questionnaire so we developed a new questionnaire for our study, which we pre-piloted, was then piloted to assess acceptability and to eliminate errors. The questionnaire which was used is included with the article (see additional file 1). It was categorised into three main sections: demographics, personal and professional experiences of weaning, and training.

An important consideration in the questionnaire (Question 2.10) was that we explicitly enquired whether the health care professional took into consideration feeding method (i.e. breast, formula or mixed) when recommending age to commence weaning. This was considered necessary as the age recommended depends on the type of milk feeing.

The data from the quantitative questionnaires was collated and analysed using the software package *Statistical Package for Social Science *(SPSS), Version 12.

### Ethics

The research proposal was submitted to the Human Research Ethics Committee of University College Dublin. The study was exempt from full ethical review as it was considered low risk. Participants of the focus group were provided with a consent form which informed them that could leave the focus group at any time, and that no identifying data would be recorded. The covering letter to potential participants in the postal survey assured them of confidentiality.

## Results

The main findings reported in this paper are concerned with knowledge of and advice given by health professionals. An additional paper looking at whether respondent's personal/demographic/attitudinal factors are associated with the type of advice given will be presented separately.

### Response Rates

194 questionnaires were returned from the sample size of 415, giving an overall response rate of 47%. Nine questionnaires were unusable as these were returned blank or with incomplete information, which left 185 (45%) available for analysis. Three health professionals returned questionnaires and excluded themselves by stating that they do not work with parents of young children. The breakdown of response rates by disciplines included GPs 42%, PNs 31%, PHNs 62% and CDs 87%.

### Knowledge of when to introduce specific foodstuffs

There was considerable variation in the response of health professionals (Table 1) overall when asked when they would advise parents to introduce specific foodstuffs. There was generally good understanding of the importance of avoiding wheat as a first food. There was considerably more uncertainty about the age of introduction of cow's milk as a main drink and the introduction of honey and nuts, fish, eggs and yoghurt.

**Table 1:** Responses given when asked appropriate age to introduce specific foods.

*Row %*	*Appropriate age*	*Early*	*On Time/Later*	*No Response*
Wheat	Over 24 weeks	10%	60%	22%

Rice	Over 17 weeks	34.5%	41.5%	16%

Cow's Milk	Over 52 weeks	16%	60%	16%

Meat	Over 17 weeks	5%	64%	23%

Poultry	Over 17 weeks	6%	63%	23%

Fish	Over 24 weeks	32.5%	39%	20.5%

Eggs	Over 24 weeks	25.5%	46.5%	20%

Yoghurt	Over 24 weeks	38.5%	33%	20.5%

Honey	Over 52 weeks	20%	25%	47%

Fruit	Over 17 weeks	61.5%	13%	17.5%

Vegetables	Over 17 weeks	59%	14%	19%

*8% of respondents indicated that they never discuss weaning

Recommended minimum age for introducing solids were based on the current guideline of minimum weaning age of 6 months for breastfed babies and 4-6 months for formula-fed babies as per ESPGHAN.

### Minimum Age to Introduce Solid Foods for breast versus bottle fed infants

**Figure 1:**
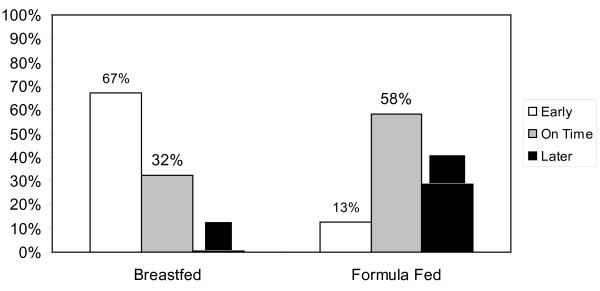
**Responses when asked Minimum Age for Weaning**. Recommended minimum age for introducing solids were based on the current guideline of minimum weaning age of 6 months for breastfed babies and 4-6 months for formula-fed babies.

The majority of respondents gave an earlier than recommended age for weaning breastfed babies (Figure 1) (67%) whereas the majority of respondents (58%) know the correct minimum age to introduce solid foods to formula fed infants.

**Figure 2:**
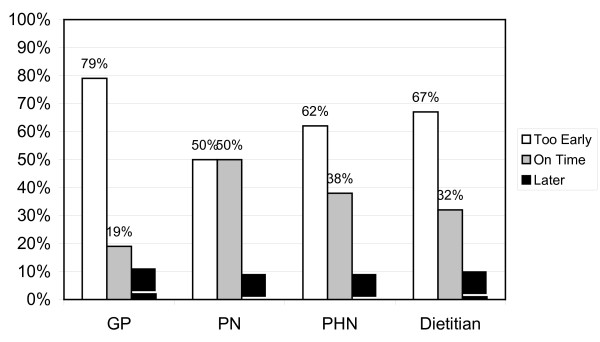
**Timing recommended by professional groups for introducing solid foods to breast-fed infants**.

**Figure 3:**
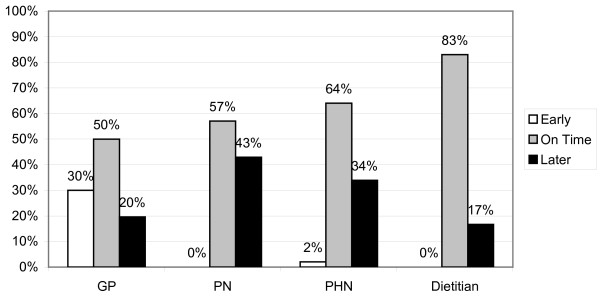
**Recommended minimum age broken down by professional group for formula fed infants**. Recommended minimum age for introducing solids were based on the current guideline of minimum weaning age of 6 months for breastfed babies and 4-6 months for formula-fed babies.

Figure 2 shows the minimum recommended age for introducing solids foods to breast-fed infants stratified by professional groupings. Figure 3 illustrates this for formula-fed infants. Particularly worrying the youngest minimum age given for the introduction of solid foods was 6 weeks. This was stated by 2 GPs and referred specifically to formula fed infants.

### Awareness of risks of early and late weaning

**Figure 4:**
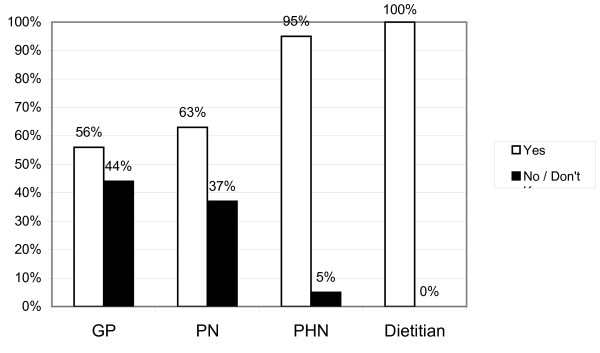
**Professionals who consider there to be risks of Early Weaning**. Recommended minimum age for introducing solids were based on the current guideline of minimum weaning age of 6 months for breastfed babies and 4-6 months for formula-fed babies.

Although 71% of respondents (n = 132) consider there to be health risks from weaning early (Figure 4), there were also sceptical responses like "Not convinced". Multiple risks are mentioned by some health professionals and risks most frequently mentioned are allergy, eczema and asthma (n = 36) food intolerance (n = 7), Celiac Disease and GI problems (n = 23), and Obesity and overweight (n = 25). PHNs and CDs are more likely to consider there to be risks from early weaning.

**Figure 5:**
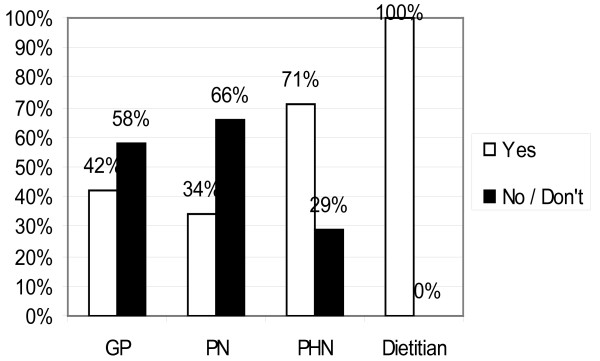
**Professionals who consider there to be risks of Late Weaning**. Recommended minimum age for introducing solids were based on the current guideline of minimum weaning age of 6 months for breastfed babies and 4-6 months for formula-fed babies.

A smaller percentage of the total number of health professionals, 49% (n = 94), consider there to be health risks from weaning late (Figure 5) with anaemia/iron deficiency being most often cited (n = 33), with other concerns being related to chewing (n = 5) poor muscle development and speech problems (n = 13), or fussiness/resistance to new tastes or lumpy foods, (n = 9).

### Centile charts

**Figure 6:**
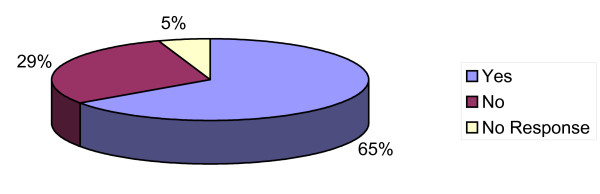
**Use of Centile Chart use amongst participants**.

Only 65% of respondents indicated that they use a centile chart for weight and growth monitoring (Figure 6), while a total of 9 different centile charts were named in regular usage.

### Parent Information Literature

**Figure 7:**
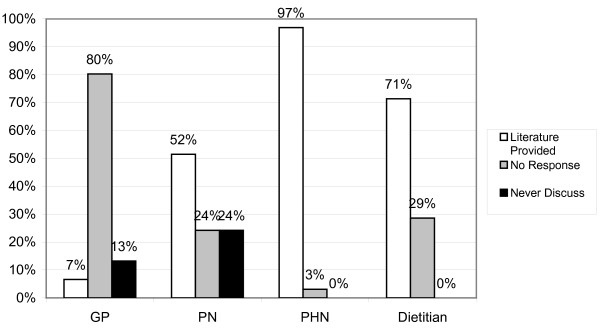
**Provision of Weaning Literature to Parents**.

The vast majority of PHNs (97%) and CDs (80%) stated that they provide weaning literature to parents (Figure 7), but only half of PNs (51.5%) and 7% of GPs provide this literature. Responses revealed that many GPs and PNs were unaware of the available literature and had a reliance on materials provided by commercial sources. The booklet *Starting to Spoonfeed Your Baby *was regularly and correctly identified by PHNs and Dieticians as the current HSE publication for parents, but was rarely mentioned by GPs and PNs.

### Vitamin supplementation

Vitamin supplementation appears to be not well understood, echoing the findings of Cleghorn [21]. There appears to be uncertainty around recommending vitamin supplements, with the vast majority of respondents (87%, n = 160) not recommending any supplementation to parents. Few respondents offered any specific recommendation although six people suggested giving Abidec (multivitamin supplements) drops if breastfeeding, for pre-maturity, or if vegetarian. No respondents mentioned caution about Abidec use with large volumes of formula milk and the consequent risk of too much Vitamin A. One PHN suggested that Vitamin D is being recommended *"recently"*, and there were two insightful remarks stating "*Only recommend if mum insists on cow's milk before one year" *and *"Awaiting advice from Health Service Executive (HSE)"*.

It is noteworthy that at the time of the study, health professionals were awaiting advice from the HSE following the publication of the document from the Food Safety Authority Ireland (FSAI) [22] which recommended that babies from birth to 12 months of age should be given 200IU (5 ug) of Vitamin D per day and stated that breastfed infants should receive Abidec supplements, while awaiting a licence for a new Vitamin D only supplement.

### Factors considered when giving advice

**Figure 8:**
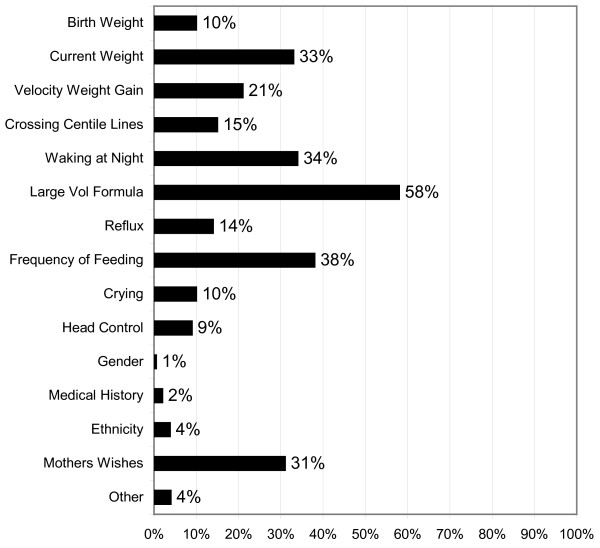
**Factors Influencing Decision to Recommend Weaning**.

Respondents were asked: "What factors other than the baby's age do you consider when giving advice about when to start weaning?" (Figure 8). The top five factors were the baby consuming a large volume of formula (58%), frequency of feeding (38%), baby waking at night (34%), current weight (33%) and the mother's wishes (31%). When respondents were asked to indicate any other factors they consider, two mentioned *"prematurity"*. Only four respondents considered the developmental readiness of the baby with descriptions: *"Acute interest in food, observing others eating" *and "*Baby's interest in watching others eat, following with eyes, opening mouth"*.

### Confidence

**Figure 9:**
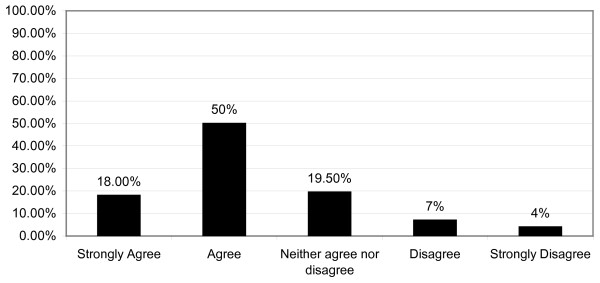
**Confidence in Giving Weaning Advice**.

A majority of respondents (68%, n = 126), felt confident in giving advice on the introduction of solid foods (Figure 9). PHNs and CDs were the more confident professional groups, with combined agreement responses of 91% and 86% respectively. GPs had the highest neither agree nor disagree and no response scores, 33% when combined. Respondents showed high levels of confidence in giving advice on the introduction of solid foods to infants, irrespective of the accuracy of the advice they provided.

### Training

**Figure 10:**
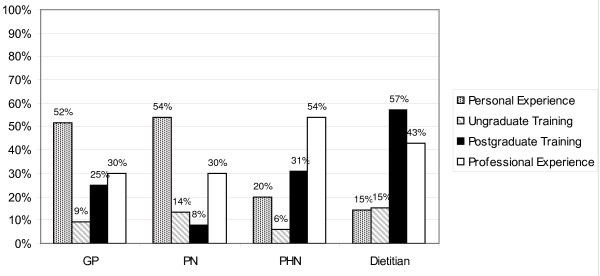
**Source of Weaning Knowledge**.

Respondents were asked what the greatest source of their weaning knowledge has been. The most usual sources of weaning knowledge were personal experience and professional experience, although CDS rated their postgraduate training highly (Figure 10). One GP felt that *"little had ever been taught about weaning"*, and another that *"the local PHN was the best source of weaning knowledge"*. A heartening finding was that PHNs who indicated that they had attended a named training module had good advice practices and high confidence scores. There were a number of additional remarks from GPs, notably: *"Couldn't choose one, includes working in the field", "Local public health nurses", "Probably use knowledge from paediatric days in hospital" *and *"Little ever said about same!" *In 28% of cases (n = 19) the PHNs mentioned postgraduate training, naming varied formats of the *"Module on food/nutrition", "HSE Days - Programme of Action for Children*", *"Training Programme for PHN and Area Medical Officer"*.

## Discussion

The timing of when solid foods should be introduced to infants, and which foods should be introduced and when, are contentious issues which appear to cause confusion for parents and professionals alike. It is likely based on our findings in this pilot study that some parents may be receiving incorrect and inconsistent advice from the very health professionals charged with informing them of optimal infant feeding practices. The study exposed varying practices, and pockets of misinformation which are of particular concern when high confidence levels were reported amongst the majority of those surveyed (68%).

Breast fed infants were being recommended to wean earlier than they should be by a majority of respondents (67%). This is clearly an area where more training may be warranted. It is interesting to note in contrast that the majority gave correct advice with respect to timing of first foods for formula fed infants. One can only assume that they are drawing from previous recommendation which once did advocate weaning at 4 months of age regardless of method of feeding as this was the official advice up to approximately 4 years ago [23] Are practitioners not keeping up to do with emerging recommendations and new literature?

With regards to "risks associated with early weaning" it is noteworthy that GPs and PNs appeared less knowledgeable about potential risks compared with CDs and PHNs. Is it a coincidence then that both the latter groups also acknowledged clearly that they play a role in providing advice to parents whereas GPs (19%) and PNs (7&) did not? This may also have been reflected in the response rates in that only 42% of GPs returned questionnaires and only 31% of practise nurses compared with high response rates from the other two professional groups surveyed.

Other issues of concern which emerged was the fact that many foodstuffs were being recommended earlier than they should have been and worryingly 35% of respondents did not make use of centile charts. Those who did report using them cited nine different ones raising issues around standardisation. Considering that this research was undertaken within a small geographical location this seems to highlight a particular training need.

Provision of literature is another area of concern with only 7% of GP's providing appropriate literature and only 50% of PNs. Reliance of GPs on commercial literature is also of concern.

Vitamin D supplementation was not recommended by 87% of respondents (however this may be explained by lack of clarity due to pending advice from the HSE).

### Strengths and limitations of the study

Since there were no existing studies on weaning advice practices in Ireland, this pilot study provides important data by seeking the views of four different professional groups providing opportunities to build on these findings within primary healthcare teams. However, the results are based on the self-reported practices, and not from audit, which may compromise reliability. The researcher accepts limitations, such as the sample being drawn from one HSE region, which may not be representative of other areas. The questionnaire was original so it had no previous validation. In addition, the response rate, especially for PNs was low.

## Conclusion

This study highlighted a number of key issues for professionals working with families, especially those who provide nutrition advice to parents. Consistent and relevant advice might make weaning messages less confusing, more realistic and acceptable, which might increase compliance to the advice thereby improving child nutrition and child health. For this to happen, parent information literature requires review to provide a consistent message, with clear guidelines written and disseminated to well-trained health professionals, with skills at imparting the information to parents in an acceptable way. This training should involve the medical, nursing and allied disciplines who regularly meet parents in the first few months after birth in primary care settings. Suitable training materials should be developed, possibly utilising e-learning techniques. Professionals should be aware of the influence of popular media and e-literature aimed at new parents, and deliver timely and appropriate weaning messages counteract them that are based on current best practice and on evidence from the scientific literature.

### Recommendations

The findings of this study show that there are varied levels of knowledge and advice practices amongst Irish Health Professionals with respect to infant weaning. The implications are wide-reaching, for policy and for practice, to include updating and integration of HSE parent information materials and prompt distribution of literature to reduce dependence on commercial materials. A review of strategies for promoting good nutrition is required, and greater clarity of guidelines on allergenic foods, Vitamin supplementation and standardisation of growth monitoring and centile charts.

Targeted training of health professionals working with young families is indicated with appraisal of infant nutrition training at undergraduate and postgraduate levels. This training should include practical skills such as health promotion, using centile charts and supporting parents to observe signs of developmental readiness in the infant.

Further research is now required since this study identified a number of research gaps including the absence of a national infant feeding survey, and a lack of studies examining professional knowledge, attitudes and advice practices about weaning. The questionnaire, original to this study, might now be used in other areas. It might also be refined for use with other health disciplines that have opportunities to provide infant nutrition advice, most particularly paediatricians, paediatric nurses and pharmacists.

## Competing interests

The authors declare that they have no competing interests.

## Authors' contributions

CA had the original idea for the study. She contributed to the study design, and collected and analysed the data. She was involved in drafting the manuscript and revising it for intellectual content.

MRS had substantial contributions to the conception and design of the study. She was also involved in drafting the manuscript and revising it for intellectual content.

Both authors read and approved the manuscript.

## Pre-publication history

The pre-publication history for this paper can be accessed here:

http://www.biomedcentral.com/1471-2458/10/201/prepub

## Supplementary Material

Additional file 1 **Questionnaire**. This file contains the questionnaire which was used in the study. Click here for file
