# Supplementary material for: An exploration of knowledge, attitudes and advice given by health professionals to parents in Ireland about the introduction of solid foods. A pilot study
Source: BMC Public Health. 2010 Apr 21;10:201. doi: 10.1186/1471-2458-10-201 (PMC2868001; doi:10.1186/1471-2458-10-201)
Supplement: Additional file 1 — Questionnaire. This file contains the questionnaire which was used in the study. [file 1471-2458-10-201-S1.DOC]

**Questionnaire used.**

**Please tick to indicate your answer. Write further comments where applicable.**

**Demographics and Personal Experience**

| 1.1 | What is your profession? | 🞏 General Practitioner  🞏 Practice Nurse  🞏 Public Health Nurse  🞏 Dietitian |
| --- | --- | --- |
| 1.2 | How many years have you been working in your present professional role? | 🞏 0-5 🞏 6-10  🞏 11-15 🞏 16 – 20  🞏 21-25 🞏 over 26 |
| 1.3 | What is your gender? | 🞏 Male 🞏 Female |
| 1.4 | Were you trained for your present professional role in Ireland? | 🞏 Yes 🞏 No |
| 1.5 | Have you worked in your present professional role in any country other than in Ireland? | 🞏 Yes  🞏 No  If yes, which country? …............................................. |
| 1.6 | Are you a parent? | 🞏 Yes 🞏 No |
| 1.7 | How were your children fed as in the first six weeks of life? | 🞏 Not Applicable  🞏 Breastfed 🞏 Formula-fed  🞏 Mixed breast- and formula-fed |
| 1.8 | When did you introduce solid foods with your own children? | 🞏 Not Applicable  1^st^ child, age in weeks  2^nd^ child, age in weeks  3^rd^ child, age in weeks  4^th^ child, age in weeks |
| 1.9 | What was the first solid food you offered your own children? | 🞏 Not Applicable  1^st^ child …………………….  2^nd^ child …………………….  3^rd^ child …………………….  4^th^ child …………………….. |
| 1.10 | Please tick how you feel about the statement: ***“My personal experience of weaning influences the advice I give to parents”*** | 🞏 Not Applicable  🞏 Strongly Agree  🞏 Agree  🞏 Neither Agree nor Disagree  🞏 Disagree  🞏 Strongly Disagree |

Professional Experience of Weaning

| 2.1 | Do you consider that discussing weaning is part of your professional role? | 🞏 No, it is not part of my role  🞏 Yes, I routinely raise the topic  🞏 Yes, but only if a parent asks  🞏 Yes, if parents are referred to me for weaning advice |
| --- | --- | --- |
| 2.2 | How many times a week would you typically discuss weaning? | 🞏 Never    🞏 Rarely 🞏 0-5 times  🞏 6-10 times ⁪ 🞏 11- more times |
| 2.3 | Who or what do you believe most influences a first-time mother regarding the introduction of solid foods?  Please tick the **THREE** that you consider most important | 🞏 Her partner ⁭🞏 Her friends⁭  🞏 Her mother ⁭🞏 Books  🞏 Her family 🞏 Internet  🞏 Her level of education  🞏 Magazines 🞏 Media  🞏 Practice Nurse 🞏 GP  🞏 PHN ⁭ 🞏 Dietitian  🞏 Paediatrician⁭ 🞏Area Medical Officer  🞏 Other…………………. |
| 2.4 | At which infant age or surgery, clinic or home visit do you usually discuss weaning? | 🞏 I never discuss weaning  🞏 Soon after birth  🞏 2 weeks 🞏 6 weeks ⁪  🞏 2 months 🞏 3 months  🞏 4 months 🞏 Other |
| 2.5 | Who do you most usually advise or discuss weaning with? | 🞏 I never discuss weaning  🞏 Mother 🞏 Father  🞏 Other family member  🞏 Don’t know |
| 2.6 | Do you usually advise or discuss weaning with each baby or just first-time? | 🞏 I never discuss weaning  🞏 First baby  🞏 Each baby |
| 2.7 | Would you like to have a specific visit or consultation to discuss weaning? | 🞏 Yes 🞏 No  If yes, please specify when you feel that it would be most effective ………………………………… |
| 2.8 | Are you aware of any published official national guidelines regarding weaning? | 🞏 Yes 🞏 No  If yes, please specify name of publication ……………………………………….. |
| 2.9 | Name any weaning or complementary infant feeding literature you give to parents | 🞏 I never discuss weaning  🞏 The literature I give is ……….…….………………………….  ………………………………………… |
| 2.10 | At what approximate age (infant’s age in weeks) do you recommend solid foods be introduced?  Please also state a minimum age if this differs from the age you prefer to recommend. | Age in weeks for a baby who is exclusively breastfed? Minimum?  Age in weeks for baby who is formula-fed? Minimum?  Age in weeks for a baby who is mixed milk feeding (breastfed and formula-fed)? Minimum? |
| 2.11 | What factors other than the baby’s age do you consider when giving advice about when to start weaning?  Please tick the **three** factors that you consider most important | 🞏 I never discuss weaning    🞏 Baby’s birth weight  🞏 Baby’s current weight  🞏 Baby’s velocity of weight gain  🞏 Crossing of centile lines  🞏 Baby waking at night  🞏 Baby taking large volumes of formula⁪  🞏 Reflux in the baby  🞏 Baby’s frequency of feeding  🞏 Baby’s crying  🞏 Baby’s head control  🞏 Baby’s sex  🞏 Family’s medical history  🞏 Family’s ethnicity/culture  🞏 Mother’s wishes  🞏 Other  ………………………………………. |
| 2.12 | At what age do you recommend cup-feeding be introduced? | 🞏 I never discuss cup-feeding    Infant’s age in weeks |
| 2.13 | What do you say to parents to about commercial baby foods? | 🞏 I never discuss weaning  🞏 Convenient 🞏 Expensive  🞏 Nutritious 🞏 Use family foods  🞏 Ok to use in moderation  🞏 Other………………….. |
| 2.14 | Do you use a centile chart to plot baby’s weight? | 🞏 Yes 🞏 No  If yes, please specify which chart  …………………………………. |
| 2.15 | Do you recommend vitamin supplementation for infants? | 🞏 Yes 🞏 No  If yes, please specify what and when  ……………………………………. |
| 2.16 | What food type do you suggest might be introduced as a first food? | 🞏 I never discuss weaning  🞏 Cereals 🞏 Fruits  🞏 Vegetables 🞏 Meat  🞏 Eggs 🞏 Rusks  🞏 Other………………….. |
| 2.17 | For each of these foods, what approximate infant’s age (in weeks) do you suggest to parents that these foods may be introduced? | 🞏 I never discuss weaning  Wheat-based cereals  Rice-based cereals  Pasteurised Cow’s milk  Red meat Poultry  Fish Eggs  Yoghurt Honey  Fruit Vegetables  Nuts Rusks |
| 2.18 | Do you consider there to be health risks from weaning early? | 🞏 Yes 🞏 No 🞏 Don’t know  If yes, please specify risks  …………………………………… |
| 2.19 | Do you consider there to be health risks from weaning late? | 🞏 Yes 🞏 No 🞏 Don’t know  If yes, please specify risks  …………………………………… |

**Training**

| 3.1 | In the last two years, how have you acquired knowledge on weaning? | 🞏 Professional journals  🞏 HSE leaflets  🞏 Infant formula manufacturers’ leaflets 🞏 Infant formula manufacturers’ events  🞏 Internet  🞏 General media  🞏 Child Nutrition Panel  🞏 Discussions with colleagues  🞏 HSE Study Days (please specify)  ……………………………..  🞏 Other (please specify)  ………………………………….. |
| --- | --- | --- |
| 3.2 | What has been the greatest source of your infant weaning knowledge?  Please tick the **ONE** factor that you consider most important | 🞏 Personal experience ⁪  🞏 Undergraduate training  🞏 Postgraduate training  🞏 Professional experience ⁪  🞏 Other (please specify)  …………………………………….. |
| 3.3 | Please tick how you feel about the statement: ***“I feel confident to give advice on the introduction of solid foods to infants”*** | 🞏 Strongly Agree  🞏 Agree  🞏 Neither Agree nor Disagree  🞏 Disagree  🞏 Strongly Disagree |

**Thank you for taking the time to complete this questionnaire**
